# Screening and functional validation of lipid metabolism-related lncRNA-46546 based on the transcriptome analysis of early embryonic muscle tissue in chicken

**DOI:** 10.5713/ab.21.0440

**Published:** 2022-01-21

**Authors:** Ruonan Chen, Kai Liao, Herong Liao, Li Zhang, Haixuan Zhao, Jie Sun

**Affiliations:** 1 College of Animal Science and Technology, Shihezi University, Shihezi, 832000, China; 2 College of Pharmacy, Shihezi University, Shihezi, 832000, China; 3 College of Medical, Shihezi University, Shihezi, 832000, China

**Keywords:** 1-Acylglycerol-3-phosphate-O-acyltransferase 2, Chicken, Lipid Metabolism, lncRNA-ENSGALT00000046546, RNA Sequencing v

## Abstract

**Objective:**

The study was conducted to screen differentially expressed long noncoding RNA (lncRNA) in chickens by high-throughput sequencing and explore its mechanism of action on intramuscular fat deposition.

**Methods:**

Herein, Rose crown and Cbb broiler chicken embryo breast and leg muscle lncRNA and mRNA expression profiles were constructed by RNA sequencing. A total of 96 and 42 differentially expressed lncRNAs were obtained in Rose crown vs Cobb broiler chicken breast and leg muscle, respectively. lncRNA-ENSGALT00000046546, with high interspecific variability and a potential regulatory role in lipid metabolism, and its predicted downstream target gene 1-acylglycerol-3-phosphate-O-acyltransferase 2 (*AGPAT2*), were selected for further study on the preadipocytes.

**Results:**

lncRNA-46546 overexpression in chicken preadipocyte 2 cells significantly increased (p<0.01) the expression levels of *AGPAT2* and its downstream genes diacylglycerol acyltransferase 1 and diacylglycerol acyltransferase 2 and those of the fat metabolism-related genes peroxisome proliferator-activated receptor γ, CCAAT/enhancer binding protein α, fatty acid synthase, sterol regulatory element-binding transcription factor 1, and fatty acid binding protein 4. The lipid droplet concentration was higher in the overexpression group than in the control cells, and the triglyceride content in cells and medium was also significantly increased (p<0.01).

**Conclusion:**

This study preliminarily concludes that lncRNA-46546 may promote intramuscular fat deposition in chickens, laying a foundation for the study of lncRNAs in chicken early embryonic development and fat deposition.

## INTRODUCTION

Rose-crown (RC) chickens are a cultivated Chinese chicken breed that is well known for its very large comb (the RC breed is introduced in Supplementary Figure S1). Cobb broiler (CB) chickens are a large, fast-growing commercial broiler breed, and its weight can reach 3 kg at the age of 6 weeks [1]. Compared with CB chickens, RC chickens exhibit a slower growth cycle but have more delicious meat and are thus more favored by consumers. Meat quality is influenced by multiple factors, such as genetic, nutritional and environmental factors, among which genetic differences play the main role [2]. Therefore, it is of great significance and commercial value to study methods for improving chicken quality on the basis of genetic differences.

Intramuscular fat (IMF) is an important factor affecting meat quality that is distributed in muscle and muscle fiber tissues [3]. IMF deposition can simultaneously promote the separation of muscle fiber bundles and improve muscle tenderness by loosening the cross-links among muscle fibers, fat and connective tissue [4]. Increased fat content contributes to better meat flavor while improving tenderness and juiciness, particularly when it occurs as IMF at levels higher than 2.5% [5]. Previous studies have confirmed that the IMF content of slow-growing chickens is significantly higher than that of fast-growing broilers in the late growth period [6]. The number of fat cells in animal muscle is determined during the embryonic and early developmental stages. In the late growth stage, fat deposition occurs only through increases in the fat cell volume [7]. Some studies of chicken embryos have shown that from embryonic day 17 (E17) to postnatal day 1, IMF is rapidly deposited in muscles but that the amount of IMF in muscle decreases sharply during later development [8]. Thus, embryonic muscle development and IMF deposition have critical effects on meat quality and meat production.

Long noncoding RNAs (lncRNAs) are transcripts longer than 200 nucleotides that lack a protein-coding ability but some can encode small peptides [9]. Previous studies focused on fat metabolism revealed that lncRNAs potentially regulate multiple biological processes, such as preadipocyte differentiation, fat cell differentiation and IMF deposition [10]. The intramuscular fat-associated long non-coding RNA (lncRNA *IMFNCR*) acts as a molecular sponge to bind to miR-128-3p and miR-27b-3p, thereby increasing the expression of the peroxisome proliferator-activated receptor γ (*PPARγ*) and promoting fat cell differentiation and IMF deposition in chicken muscle [11]. Additionally, the lncAD has been shown to inhibit thioredoxin reductase 1 (*TXNRD1*) expression in a cis-regulatory manner and to decrease intramuscular preadipocyte adipogenic differentiation and promote cell proliferation [12]. However, little is known about the expression profiles of lncRNAs related to IMF deposition in early embryonic development.

In the study, lipid droplets in embryonic muscle tissue of two chicken with different genetic backgrounds at 7 and 8 days were stained with oil red, and the triglyceride content in muscle at 42 days after hatching was compared. Differentially expressed lncRNAs and mRNAs were identified from breast and leg muscles of two chicken embryos. We then analyzed the effect of lncRNA-ENSGALT00000046546 (lncRNA-46546) on immortalized chicken preadipocyte 2 (ICP2) cells (College of Animal Science and Technology, Northeast Agricultural University, China) fat metabolism and proliferation [13]. This paper provides a valuable base for further studies on the molecular mechanism underlying chicken IMF deposition, leading to a better understanding of the biological process.

## MATERIALS AND METHODS

### Ethical statement

All experimental animals were handled according to a protocol approved by the Medical Ethics Committee of the First Affiliated Hospital, Medical College, Shihezi University (A2016-095, 9 March 2016). All animal experiments were in line with the Guide for the Care and Use of Laboratory Animal by International Committees.

### Sample preparation

Two chicken breeds, RC and CB, were subjected to high-throughput sequencing. One hundred fertilized RC and CB chicken eggs were incubated at 37°C under 60% humidity, and E7 leg muscles (L) and E8 breast muscles (B) were surgically collected on a clean bench, immediately placed in liquid nitrogen and then stored at −80°C. The embryonic brain was collected for sex identification by referencing the protocol of Vucicevi et al [14]. Male samples were divided into four groups, rose-crown chicken leg muscles (RCL), Cobb broiler chicken leg muscles (CBL), rose-crown chicken breast muscles (RCB), and Cobb broiler chicken breast muscles (CBB), for high-throughput sequencing, with 3 replicates of each group. At 42 days after hatching, breast and leg muscles were sampled from the two chicken breeds and stored at −80°C for the determination of IMF and triglyceride (TG) contents in 3 replicates.

### Determination of tissue intramuscular fat content and oil red O staining

Intramuscular fat determination was performed in 10 g samples of breast and leg muscle. After the samples were thawed at 4°C for 48 h, adherent adipose and connective tissue were removed from the muscle and then freeze-dried overnight. Petroleum ether fat extraction from the resultant dried product was conducted for 8 h using a Soxtec Extraction System, and the extracted fat was then dried for 1 h at 105°C. The IMF content was expressed on a freeze-dried basis.

Fresh and equally sized breast (E8) and leg (E7) muscle tissues were collected for compression slices. After slicing and fixing, compression slices were stained with oil red O and hematoxylin. The staining results were observed with a light microscope.

### RNA library construction and transcriptome assembly

Total RNA was extracted using TRIzol Reagent (Invitrogen, Carlsbad, CA, USA) according to the manufacturer’s instructions. The purity, concentration, and integrity of the total RNA were checked using a Nano Photometer spectrophotometer (IMPLEN, Munich, Germany), a Qubit 2.0 Fluorometer (Life Technologies, Carlsbad, CA, USA), and a RNA Nano 6000 Assay Kit with a Bioanalyzer 2100 system (Agilent Technologies, Santa Clara, CA, USA), respectively. The sequencing libraries were generated from rRNA-depleted RNA with the NEBNext Ultra Directional RNA Library Prep Kit for Illumina (NEB, Boston, MA, USA). After cluster generation with a TruSeq PE Cluster Kit v3-cBot-HS (Illumina, USA), the libraries were sequenced on the Illumina HiSeq 4000 platform at the Novogene Bioinformatics Institute (Beijing, China), and 150 bp paired-end reads were generated. Clean reads were obtained by removing reads containing adapters, reads containing poly-N sequences and low-quality reads from the raw data. The chicken reference genome and gene model annotation files were downloaded from the Ensembl genome browser (Ensembl Release 90, *Gallus gallus* 5.0, http://asia.ensembl.org/Gallus_gallus/Info/Index). The reference genome index was built using HISAT2-build (v2.0.4), and paired-end clean reads were aligned to the reference genome using HISAT (v2.0.4) with “--rna strandness RF” and other parameters set to the defaults [15]. The mapped reads of each sample were assembled by using StringTie (v1.3.1) [16].

### LncRNA identification

The transcriptome splicing results were based on the structural characteristics of lncRNAs and the functional characteristics of noncoded proteins. First, we used Cuffmerge software to merge the transcripts and removed the transcripts whose chain direction was uncertain. The identified transcripts were then screened via the following 5-step screening process, and the selected lncRNAs were used as the final candidate lncRNA set for subsequent analysis: i) transcripts with ≥2 exons were selected; ii) transcripts with a length >200 bp were selected; iii) Cuffcompare (v2.1.1) software was used to screen out transcripts that overlapped with the exon region of the database annotation, which were used for lncRNA annotation in the subsequent analysis; iv) the expression level of each transcript was calculated with Cuffquant software, and transcripts with ≥0.5 fragments per kb of transcript per million mapped reads (FPKM) were selected; finally, v) three software programs were used for coding potential analysis to identify lncRNAs: Pfam-sca (E-value<0.001, v1.3), CPC2 (score<0, v0.1), and CNCI (score<0, v2). The intersection of the results of the three programs was used as the lncRNA dataset predicted by this analysis.

### Differential expression and conservation analyses

Differential expression was determined from the digital transcript or gene expression data using a model based on the negative binomial distribution [17]. In the RCB vs CBB and RCL vs CBL comparisons, transcripts with a Q-adjusted value<0.05 were identified as differentially expressed. To calculate the sequence conservation of transcripts, two programs in Phast (v1.3) were used: phyloFit and phastCons (v1.3) [18]. phyloFit was used to compute phylogenetic models for conserved and nonconserved regions among species and was run with the parameter --tree “(mm10, (galGal4, hg19))”; then, the model and hidden Markov model transition parameters were used to compute a set of conservation scores of lncRNA and coding genes with phastCons.

### Target gene prediction and functional enrichment analyses

To explore the function of lncRNAs, we first predicted the cis and trans target genes of the lncRNAs. Cis activities refer to lncRNAs acting on neighboring target genes. The coding genes located 10 kb–100 kb upstream and downstream of each lncRNA were searched. The trans function of target gene prediction for lncRNAs is to identify lncRNAs by their expression levels using the Pearson correlation coefficient (|r|>0.95) as a final result. Gene ontology (GO) enrichment analyses of differentially expressed genes or lncRNA target genes were implemented with the GO seq R package (Release 2.12) [19]. Differentially expressed lncRNA genes were statistically enriched in Kyoto encyclopedia of genes and genomes (KEGG) pathways by using KOBAS (v2.0) software [20]. GO terms and KEGG pathways with corrected p-values<0.05 were considered significantly enriched in differentially expressed genes.

### Rapid amplification of cDNA ends

Rapid amplification of cDNA ends (RACE) polymerase chain reaction (PCR) was performed to obtain the full-length sequence of lncRNA-46546. Total RNA from breast muscle tissue was employed as the template for nested PCR using a SMARTer RACE cDNA Amplification Kit (Takara, Tokyo, Japan) following the manufacturer’s instructions. The RACE PCR products were cloned into the pUC19 vector (Takara, Japan) and sequenced by Sangon Biotech (Shanghai, China).

### Primers and small interfering RNAs

Primers were designed using Premier Primer 5.0 software (Premier Biosoft International, Palo Alto, CA, USA) and synthesized by Sangon Biotech (China). The *U6* small nuclear RNA and glyceraldehyde-3-phosphate dehydrogenase genes were selected as reference genes. The quantitative real-time PCR (qRT-PCR) primer information is provided in the supplemental material (Supplementary File S1). The primers used for cloning the full-length chicken lncRNA-46546 are shown in Table 1. Among these primers, lncRNA-46546-5′RACE-outer and lncRNA-46546-5′RACE-inner were used to clone the lncRNA 5′ sequence, lncRNA-46546-3′RACE-outer and lncRNA-46546-3′RACE-inner were used to clone the lncRNA 3′ sequence, and lncRNA-46546-5 and lncRNA-46546-3 were used to amplify the full-length sequence. The PCR products were subsequently excised with the *KpnI* and *XhoI* restriction endonucleases and ligated into the pcDNA3.1(+) plasmid vector. The overexpression vector was named pcDNA3.1(+)-46546. The small interfering RNAs (siRNAs) used for the specific knockdown of lncRNA-46546 were designed and synthesized by GenePharma (Shanghai, China) and are listed in Table 2.

### Cell culture and transfection

ICP2 cells were obtained from Northeast Agricultural University. The ICP2 cells were cultured in DMEM/F12 medium (Gibco, Carlsbad, CA, USA) supplemented with 10% fetal bovine serum (Biological Industries, Kibbutz Beit Haemek, Israel) plus 100 units/mL penicillin and 100 μg/mL streptomycin (Gibco, USA) at 37°C under 5% CO_2_. The transfection reactions were performed using the Lipofectamine 2000 reagent (Invitrogen, USA) in Opti-MEM (Gibco, USA) following the manufacturer’s instructions. Harvested cells were analyzed 48 h after transfection.

### Cell oil red O staining and triglyceride content determination

Cells were washed with phosphate-buffered saline (PBS) and stained using an oil red O Stain Kit (Solarbio, BeiJing, China) following the manufacturer’s instructions. The cells were then observed and photographed with an inverted fluorescence microscope.

After harvesting the cells, an appropriate amount of PBS was added to resuspend the cells, which were then subjected to ultrasonic cell disruption at 130 W power 10 times, for 8 to 10 s each time at 15 s intervals, followed by centrifugation at 4°C and 12,000 rpm for 5 min. Finally, the supernatant was collected as the protein sample. The protein content was determined using the bicinchonininc acid method. TG assay working fluid (Nanjing Jiancheng, Nanjing, China) was mixed with the protein, or with ddH2O as a blank control, followed by incubation at 37°C for 10 min, and the optical density (OD) was then determined at a 510-nm wavelength. Moreover, cell culture medium was collected to measure TG contents using the TG content assay kit of Beijing Boxbix Science &b Technology Co., Ltd. (Beijing, China) Standard and blank wells were included to measure the OD at 420 nm wavelength.

### CCK-8 assay

After counting the cells, they were seeded in a 96-well plate. When the cells reached a density of 50% to 60%, they were transfected with pcDNA3.1(+)-46546, pcDNA3.1(+), siRNA, and siRNA-NC, and a blank control was also set up, each with 3 replicates. The proliferation of the cells was monitored at 24, 48, 72, and 96 h using a Cell Counting Kit-8 (Dojindo, Kyushu, Japan). Every 24 h, CCK-8 solution was added to the medium, and the OD at 450 nm was determined using an enzyme-labeled instrument after incubation for 1 h.

### qRT-PCR verification

Total RNA was reverse transcribed using the PrimeScript RT reagent Kit with gDNA Eraser (TaKaRa, Japan), and qRT-PCR was performed using a LightCycler 96 (Roche, Basel, Switzerland). Each 20 μL reaction contained 10 μL of LightCycler 480 SYBR Green I Master Mix (Roche, Switzerland), 7 μL of ddH_2_O, 1 μL of cDNA, 1 μL of a specific forward primer (10 pmol/μL) and 1 μL of a specific reverse primer (10 pmol/μL). The reaction conditions were as follows: preincubation at 95°C for 5 min, followed by denaturation at 95°C for 10 s, annealing at the optimal temperature for 20 s, and extension at 72°C for 10 s for a total of 45 cycles, and then a final incubation at 95°C for 5 s, 65°C for 1 min, and 97°C for 1 s for melting curve analysis. Gene expression levels were normalized using the 2^−ΔΔCT^ method. Differential expression analysis was performed via one-way analysis of variance with SPSS 22.0 software, and p<0.05 was defined as indicative of a significant difference.

### Statistical analysis

The experimental data were collated and analyzed by EXCEL software, and the fluorescence quantitative PCR results were calculated by 2^−ΔΔCT^ method. SPSS 22.0 software was used for one-way analysis of variance or independent sample T test, Duancan’s method for significance test, and Pearson method for correlation analysis. Results are expressed as “mean±standard deviation”.

## RESULTS

### Oil red O staining and IMF and TG content determination

**Figure 1:**
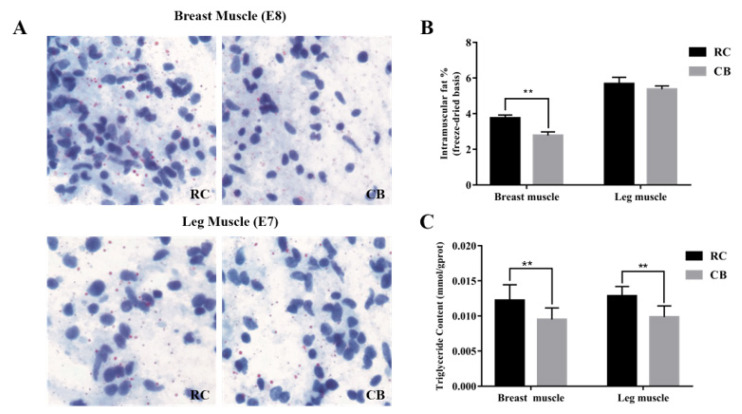
Oil red O staining and determination of IMF and TG contents. (A) Oil red O staining of compression slices of RC and CB breast (E8) and leg muscle (E7) tissue. Lipids were dyed red by oil red O, and nuclei were dyed blue by hematoxylin. Magnification: 40× (B) Comparison of IMF contents in breast and leg muscles between the two chicken breeds. ** Denotes p<0.01. (C) Comparison of TG contents in breast and leg muscles between the two chicken breeds. ** Denotes p<0.01. * Denotes p<0.05. IMF, intramuscular fat; TG, triglyceride; RC, rose-crown; CB, Cobb broiler.

Lipid deposition in the breast muscles of RC embryos was greater than that in CB embryos on E8, whereas lipid deposition in the leg muscles in RC embryos differed less from that in CB embryos on E7 (Figure 1A). At 42 days after hatching, the IMF content of RC breast muscle was significantly higher than that of CB breast muscle; the IMF content of RC leg muscle was also higher than that of CB leg muscle, but not significantly so (Figure 1B). The results of TG content determination showed that contents of RC breast and leg muscles were significantly higher than those of CB muscles (Figure 1C).

### Sequencing results and identification of lncRNAs and mRNAs

According to the analysis of the RNA-seq results of RCB, CBB, RCL and CBL (3 replicates in each group), the number of raw reads ranged from 91,374,036 to 128,934,028. The Q20 values ranged from 96.27% to 98.18%, the Q30 values ranged from 90.6% to 95.12%, and the GC concentration ranged from 45.25% to 50.17%. These results showed that the sequencing data from all 12 samples met the requirements for subsequent analysis.

**Figure 2:**
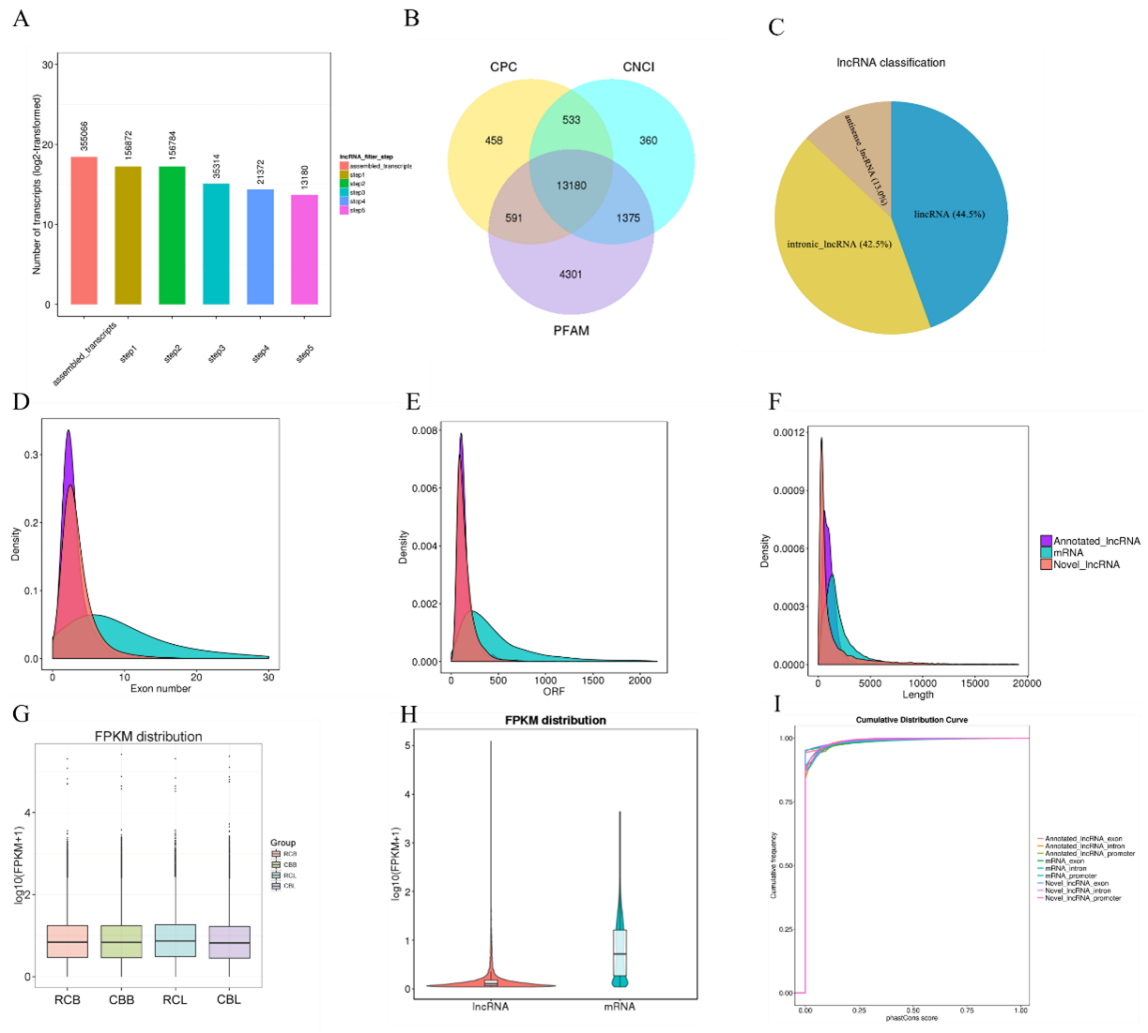
Screening and characteristics of lncRNAs and mRNAs. (A) A total of 355,066 transcripts were assembled by using Cufflinks with a stringent filtering pipeline to discard transcripts without all the characteristics of lncRNAs. (B) Identification of lncRNAs by using CPC, CNCI, and PFAM. A total of 13,180 transcripts were identified by the three software programs, and both protein-coding transcripts and putative protein-coding transcripts were removed. (C) Classification of novel lncRNAs. (D) Exon numbers, (E) ORF length distributions and ORF sequences of lncRNAs predicted by EMBOSS: getorf. (F) mRNA, annotated lncRNA and novel lncRNA lengths. (G) Box plot of the expression levels of lncRNAs in four libraries (shown in log10 (FPKM+1)). (H) Violin plot of the expression levels of mRNAs and lncRNAs (shown in log10 (FPKM+1)). (I) Conservation scores of mRNAs, annotated lncRNAs and novel lncRNAs. ORF, open reading frames; FPKM, fragments per kb of transcript per million mapped reads.

A total of 355,066 assembled transcripts were obtained by splicing and merging the total mapped transcripts (Figure 2A). Through comparison with the Ensembl database, a total of 1,100 annotated lncRNAs were identified, including 1,095 long intergenic noncoding RNAs (lincRNAs) and 5 miscellaneous RNAs (miscRNAs). A total of 13,180 noncoding transcripts (Figure 2B) were identified with the three screening software programs (Pfam-sca, CPC and CNCI), comprising 5,867 lincRNAs (44.5%), 5,606 intronic lncRNAs (42.5%) and 1,707 antisense lncRNAs (13.0%) (Figure 2C). In addition, 30,252 annotated mRNAs were identified.

### Comparison of lncRNA and mRNA characteristics

By comparison, we found that lncRNAs had fewer exons (Figure 2D) and shorter open reading frames than mRNAs (Figure 2E). The annotated lncRNAs and novel lncRNAs were shorter in length than the mRNAs (Figure 2F). The lncRNAs in different libraries showed similar expression levels (Figure 2G), and the expression levels of the mRNAs were higher than those of the lncRNAs (Figure 2H). The results of conservation analysis showed that the exons of the lncRNAs were more conserved and that their introns and promoters were similarly conserved to those of mRNAs (Figure 2I). These feature comparisons are consistent with the results of previous studies and confirm the accuracy of our lncRNA screening results [21].

### Analysis of differentially expressed lncRNAs and mRNAs

**Figure 3:**
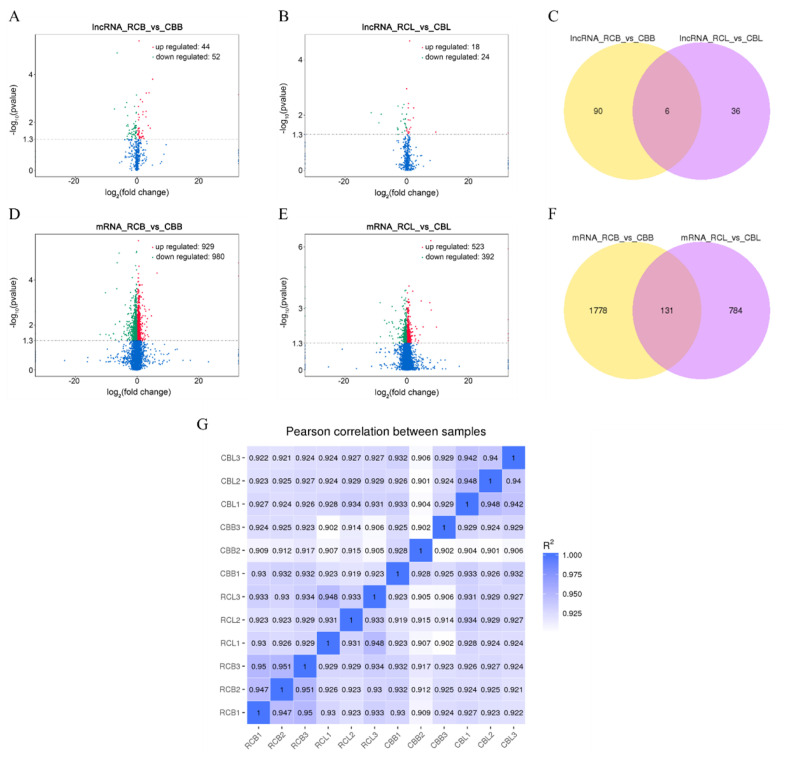
Analysis of differentially expressed lncRNAs and mRNAs. (A), (B), (D), and (E) Volcano plots of differentially expressed lncRNAs and mRNAs from two comparisons (RCB vs CBB and RCL vs CBL). (C) and (F) Venn diagram of differentially expressed lncRNAs and mRNAs from two comparisons (RCB vs CBB and RCL vs CBL). (G) Correlation map between all samples. The color range from blue to white indicates high to low correlations (RCB, rose-crown chicken breast muscles; CBB, Cobb broiler chicken breast muscles; RCL, rose-crown chicken leg muscles; CBL, Cobb broiler chicken leg muscles).

We identified a total of 132 differentially expressed lncRNAs and 2,693 differentially expressed mRNAs (p<0.05). Ninety-six lncRNAs (44 upregulated and 52 downregulated, Figure 3A) and 1,909 mRNAs (929 upregulated and 980 downregulated, Figure 3D) were differentially expressed between the RCB and CBB groups. Forty-two lncRNAs (18 upregulated and 24 downregulated, Figure 3B) and 915 mRNAs (523 upregulated and 392 downregulated, Figure 3E) were differentially expressed between the RCL and CBL groups. Six lncRNAs and 131 mRNAs were differentially expressed in RCB vs CBB and RCL vs CBL, respectively (Figure 3C, 3F).

The correlation coefficient can represent the degree of similarity among samples. We performed a Pearson correlation analysis on all samples, and the results showed that the correlation among samples was greater than 0.9, indicating a high correlation (Figure 3G). Cluster analysis results showed that all samples presented repeatable results except for CBB3 and CBL3, potentially because of individual differences.

### qRT-PCR verification

To confirm the accuracy of the RNA-seq results, we randomly selected 8 differentially expressed lncRNAs (ENSGALT000 00046546, ENSGALT00000079684, ENSGALT00000047644, ENSGALT00000081660, ENSGALT00000069012, ENSGALT 00000078880, LNC004788 and LNC012497) and 12 differentially expressed mRNAs (ribosomal protein L37a [*RPL37A*], H4 clustered histone 11 [*HIST1H4J*], heterogeneous nuclear ribonucleoprotein H1 [*HNRNPH1*], myosin light chain 2 [*MYL2*], meteorin like [*METRNL*], notch receptor 1 [*NOTCH1*], cyclin Y like 1 [*CCNYL1*], RAD51 associated protein 1 [*RAD51AP1*], refined palm olein [*ROL24*], inhibitor of DNA binding 3 [*Id3*], platelet derived growth factor receptor alpha [*PDGFRA*], and cDNA proteasome subunit 7 [cpsmb7]) for verification, each with 3 replicates. The results showed that all lncRNA and mRNA expression trends were consistent with the RNA-seq results (Supplementary Figure S2), indicating that our RNA-seq results were reliable.

### Enrichment analysis of differentially expressed mRNAs

**Figure 4:**
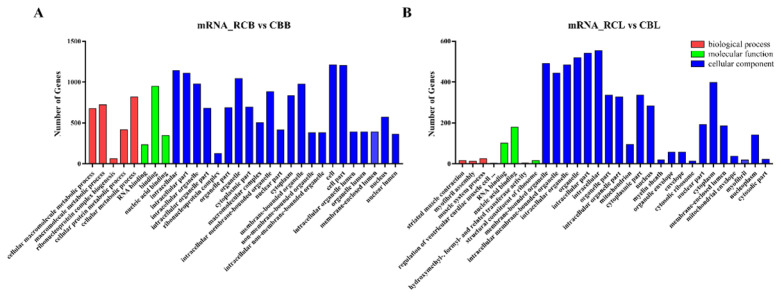
GO and KEGG analysis of differentially expressed mRNAs. (A) and (B) Histogram of the GO enrichment of differentially expressed mRNAs. Red denotes biological processes, green denotes molecular functions, blue denotes cellular components; the top 30 terms identified in the analysis are displayed. (C) and (D) Scatter plot of the KEGG enrichment of differentially expressed mRNAs. The top 20 terms identified in the analysis are displayed. GO, gene ontology; KEGG, Kyoto encyclopedia of genes and genomes.

GO analysis revealed the functions of genes in stage-specific modules, and pathway analysis revealed essential pathways and metabolic networks of genes. In this study, a total of 1,016 GO terms were significantly enriched (p<0.05) according to the GO results for the comparison between the RCB and CBB groups. A total of 471 GO terms were significantly enriched (p<0.05) according to the GO results for the comparison between the RCL and CBL groups. The significantly enriched GO terms of the mRNAs were related primarily to organelle and cell biological processes (Figure 4A, 4B). KEGG pathway analysis identified 15 significantly enriched pathways (p<0.05), including the ECM-receptor interaction, adherens junction, biosynthesis of amino acids, RNA degradation and transport, protein processing in endoplasmic reticulum and mammalian target of rapamycin (mTOR) signaling pathways (Figure 4C, 4D).

### Functional prediction of lncRNAs

**Figure 5:**
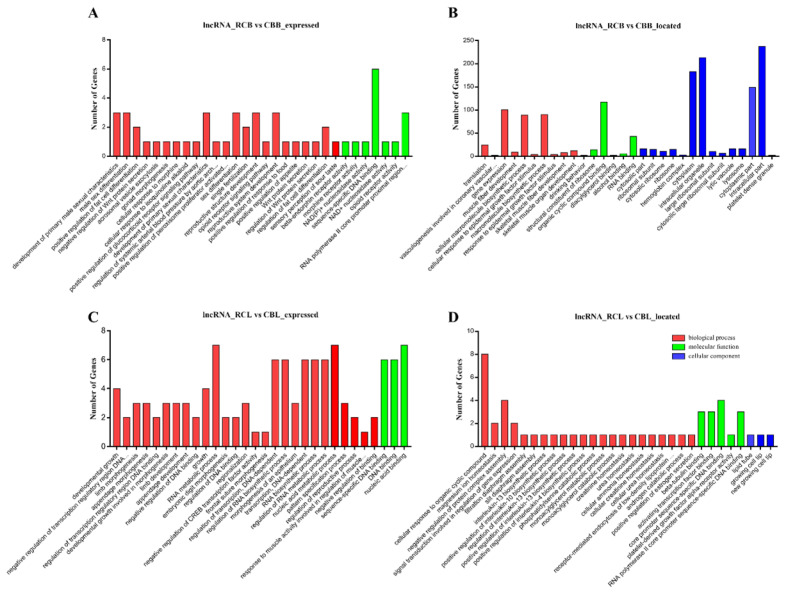
GO analysis of differentially expressed lncRNAs. (A) GO analysis of cis target genes in RCB vs CBB. (B) GO analysis of trans target genes in RCB vs CBB. (C) GO analysis of cis target genes in RCL vs CBL. (D) GO analysis of trans target genes in RCL vs CBL. Red denotes biological processes, green denotes molecular functions, blue denotes cellular components; the top 30 terms identified in the analysis are displayed. GO, gene ontology; RCB, rose-crown chicken breast muscles; CBB, Cobb broiler chicken breast muscles; RCL, rose-crown chicken leg muscles; CBL, Cobb broiler chicken leg muscles.

To assess the potential roles of lncRNAs in chicken IMF deposition and to identify key molecular participants in the process, the potential cis and trans-target genes of differentially expressed lncRNAs were predicted by bioinformatics analysis. A total of 608 candidate target genes were identified from 132 differentially expressed lncRNAs, including 566 cis target genes and 42 trans target genes. We identified fat metabolism genes among the cis target genes, such as 1-acylglycerol-3-phosphate-O-acyltransferase 2 (*AGPAT2*), *NOTCH1*, fat storage inducing transmembrane protein 2 (*FITM2*), *PDGFRA*, *Id3*, acyl-CoA synthetase bubblegum family member 2 (*ACSBG2*), transcription factor AP-2 beta (*TFAP2B*), mediator complex subunit 1 (*MED1*), ER lipid raft associated 2 (*ERLIN2*), DDHD domain containing 2 (*DDHD2*), patatin like phospholipase domain containing 2 (*PNPLA2*), and STAR related lipid transfer domain containing 3 (*STARD3*), which were located near ENSGALT00000 046546, ENSGALT00000054490, ENSGALT00000066033, ENSGALT00000081660, ENSGALT00000057666, ENSGALT 00000077852, ENSGALT00000079684, ENSGALT000000 51979, ENSGALT00000047025, ENSGALT00000056842, LNC_008241 and LNC_010807, respectively. Based on biological process analysis, differentially expressed lncRNA candidate target genes were found to be involved in the following processes: regulation of the interleukin biosynthetic process, DNA binding, limb development, embryonic digit morphogenesis, regulation of RNA metabolic process, structural constituent of the ribosome, skeletal muscle development, positive regulation of fat cell differentiation, regulation of fat cell differentiation and sex differentiation (Figure 5). In the pathway analysis, the significantly enriched pathways (p<0.05) included the following: lysosome, ribosome, gap junction, tight junction, mTOR signaling pathway, p53 signaling pathway, N-glycan biosynthesis, base excision repair, glycerolipid metabolism, phagosome, nicotinate and nicotinamide metabolism.

### RNA-seq expression level and genome location of lncRNA-46546

According to our RNA-seq results, lncRNA-46546 was differentially expressed only between the RCB and CBB groups. The expression levels of lncRNA-46546 and the *AGPAT2* gene are shown in Table 3. On chicken chromosome 17, lncRNA- 46546 is located 11.2 k-bp upstream of the *AGPAT2* gene (Supplementary Figure S3A), suggesting that the *AGPAT2* gene may be a potential cis target gene of lncRNA-46546. lncRNA-46546 has 5 exons, and its predicted sequence length is 1,337 bp. Its accession number in NCBI is LOC100858649.

### Amplification the full-length sequence of lncRNA-46546

The amplified portion of the 5′ end of the lncRNA was by 1,222 bp in length, of which 919 bp was completely consistent with the known sequence of lncRNA-46546 (Supplementary Figure S3B; Supplementary File S4A). The amplified portion of the 3′ end of the lncRNA was 1,100 bp in length, of which 924 bp coincided with the known sequence of lncRNA-46546, and there was a poly-A tail structure at the 3′ end (Supplementary Figure S3C, Supplementary File S4B). The full-length sequence of lncRNA-46546 was obtained by touchdown PCR and ligated to the pcDNA3.1(+) plasmid vector.

### Detection of overexpression and interference efficiency

**Figure 6:**
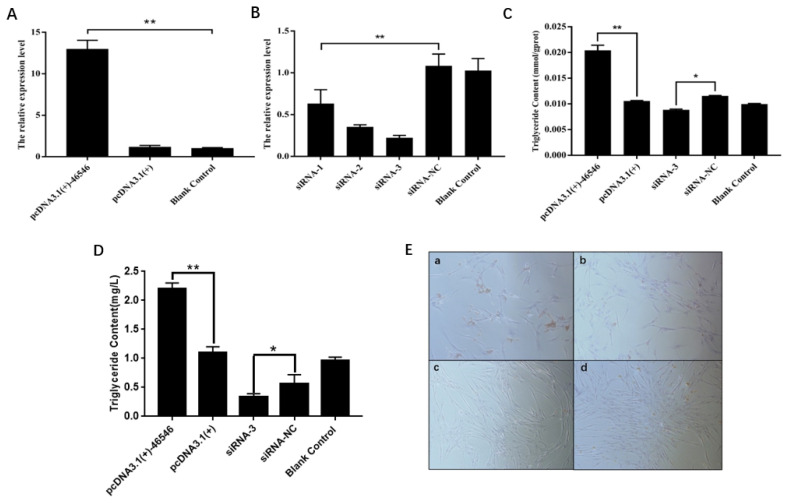
lncRNA-46546 promotes lipid deposition and TG synthesis in ICP2 cells. (A) pcDNA3.1(+)-46546 significantly increased the expression of lncRNA-46546 in ICP2 cells. (B) The expression of lncRNA-46546 was lowest after the transfection of siRNA-3. ** Denotes p<0.01. (C) lncRNA-46546 promoted TG synthesis in ICP2 cells. ** Denotes p<0.01. * Denotes p<0.05. (D) lncRNA-46546 promoted TG synthesis in ICP2 cells culture medium. ** Denotes p<0.01. * Denotes p<0.05. (E) Treatments applied to ICP2 cells: (a) pcDNA3.1(+)-46546 transfection, (b) pcDNA3.1(+) transfection, (c) siRNA-3 transfection, and (d) siRNA-NC transfection. After staining with oil red O, lipids were dyed red by oil red O, and nuclei were dyed blue by hematoxylin. Magnification, 40×. TG, triglyceride; ICP2, immortalized chicken preadipocyte 2.

ICP2 cells were transfected with the recombinant plasmid pcDNA3.1(+)-46546, the empty plasmid pcDNA3.1(+), siRNAs and siRNA-NC, with ddH2O serving as a blank control, and the expression of lncRNA-46546 was detected by qRT-PCR. Each group included three replicates. The results showed that the expression of lncRNA-46546 increased significantly after transfection with pcDNA3.1(+)-46546, reaching a level 10.96 times higher than that following pcDNA3.1(+) transfection and 12.49 times higher than that in the blank control treatment (Figure 6A). Compared with the results of siRNA-NC treatment, the interference efficiencies of the 3 siRNAs were as follows: siRNA-1, 41.7%; siRNA-2, 67.3%; and siRNA-3, 79.4%. Therefore, siRNA-3 presented the best interference efficiency and was selected for subsequent experiments (Figure 6B).

### lncRNA-46546 promotes lipid deposition and triglyceride synthesis in ICP2 cells

We first detected the effects of lipid deposition and TG synthesis after the overexpression and knockdown of lncRNA- 46546 in ICP2 cells. Each group included three replicates. The results are shown in Figure 6C. Relative to the pcDNA3.1(+) treatment, the deposition of lipids following pcDNA3.1(+) -46546 treatment was greater and denser, and the deposition of lipids following siRNA-3 and siRNA-NC treatment was relatively low. The results of TG content determination showed that after transfection with pcDNA3.1(+)-46546, the TG content of ICP2 cells was significantly increased relative to that in the other treatment groups, while the TG content of ICP2 cells was significantly decreased after the transfection of siRNA-3. Moreover, the analysis of the TG content of the cell culture medium also showed that the TG content of ICP2 cells transfected with pcDNA3.1(+)- 46546 was significantly higher than that in the other treatment groups and that the TG content of ICP2 cells transfected with siRNA-3 was significantly lower, which was very similar to our findings within cells (Figure 6D). These results indicate that lncRNA-46546 can promote the formation of lipids in ICP2 cells (Figure 6E).

### lncRNA-46546 promotes the expression of AGPAT2 and some genes

**Figure 7:**
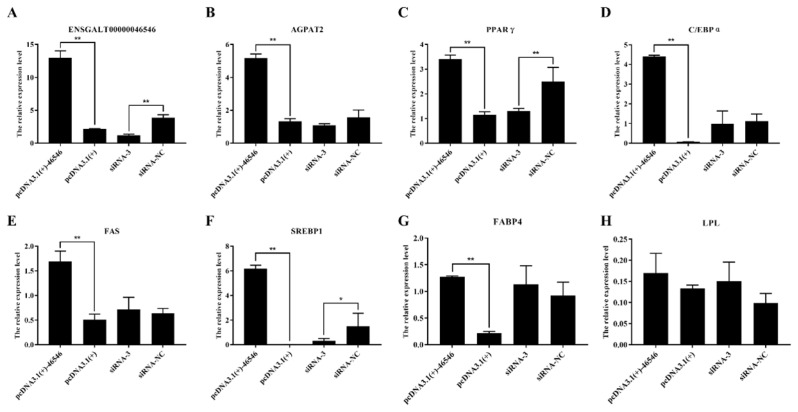
lncRNA-46546 promotes the expression of some genes and inhibits the proliferation of ICP2 cells. (A) The expression levels of lncRNA-46546 after different treatments. (B) to (G) lncRNA-46546 promoted the expression of the *AGPAT2*, *PPARγ*, *C/EBPα*, *FAS*, *SREBP1*, and *FABP4* genes. (H) lncRNA-46546 did not affect the expression of the *LPL* gene. ** Denotes p<0.01. * Denotes p<0.05. (ICP2, immortalized chicken preadipocyte 2; *AGPAT2, 1*-acylglycerol-3-phosphate-O-acyltransferase 2; *PPARγ*, peroxisome proliferator-activated receptor γ; *C/EBPα*, CCAAT enhancer binding protein alpha; *FAS*, fas cell surface death receptor; *SREBP1*, sterol regulatory element binding transcription factor 1; *FABP4*, fatty acid binding protein 4; *LPL*, lipoprotein lipase.

To explore the effect of lncRNA-46546 on mRNA expression in ICP2 cells, the expression of mRNA in the cells was detected by qRT-PCR after different treatments, each with 3 replicates. The results showed that the expression levels of the lncRNA-46546 and *AGPAT2* genes were significantly increased after the overexpression of lncRNA-46546. Conversely, the expression of lncRNA-46546 was significantly decreased, and the expression of the *AGPAT2* gene was decreased and did not reach a significant level after knockdown of lncRNA- 46546 (Figure 7A, 7B). According to these results, lncRNA- 46546 can promote rather than inhibit the expression of the *AGPAT2* gene. We preliminarily identified *AGPAT2* as the cis target gene of lncRNA-46546.

**Figure 8:**
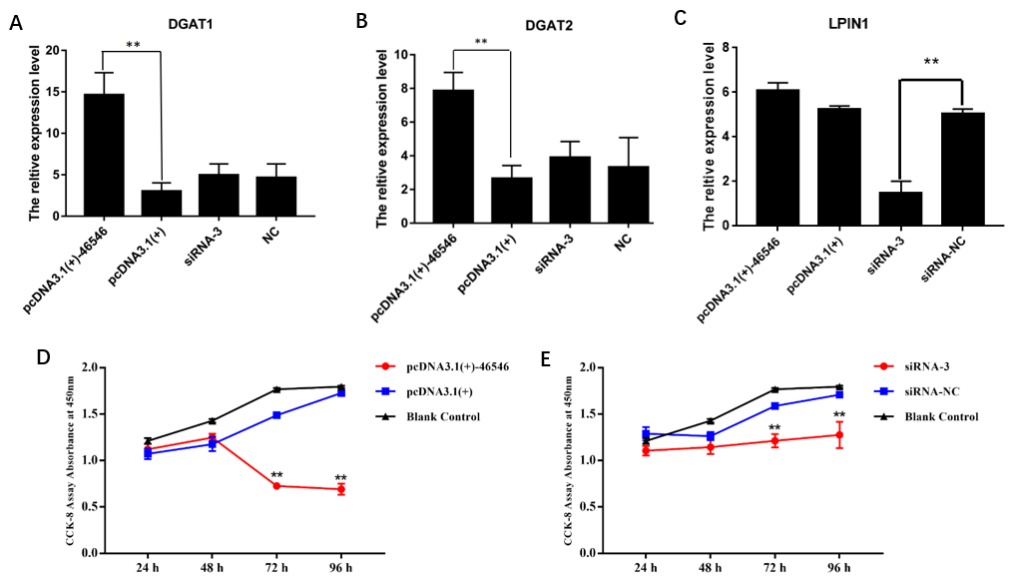
(A) to (C) lncRNA-46546 promoted the expression of the *DGAT1*, *DGAT2*, and *LPIN1* genes. ** Denotes p<0.01. (D) lncRNA-46546 significantly inhibited the proliferation of ICP2 cells. (E) Knockdown of lncRNA-46546 had a weaker effect on the proliferation of ICP2 cells. ** Denotes p<0.01. * Denotes p<0.05. *DGAT1*, diacylglycerol acyltransferase 1; *DGAT2*, diacylglycerol acyltransferase 2; *LPIN1*, lipid phosphate phosphohydrolase 1; ICP2, immortalized chicken preadipocyte 2.

Then, we also detected the expression of several classical genes closely related to lipid metabolism, and the results are shown in Figure 7. The expression levels of the *PPARγ*, CCAAT enhancer binding protein alpha (*C/EBPα*), fas cell surface death receptor (*FAS*), sterol regulatory element binding transcription factor 1 (*SREBP1*), and fatty acid binding protein 4 (*FABP4*) genes were significantly increased after the overexpression of lncRNA-46546. After the knockdown of lncRNA- 46546, the expression levels of the *PPARγ* and *SREBP1* genes were significantly decreased, but the expression level changes in the *C/EBPα*, *FAS*, and *FABP4* genes were not significantly different. The changes in lipoprotein lipase (*LPL*) gene expression were not significantly different between the treatments. We also examined the changes in the gene expression levels of diacylglycerol acyltransferase 1 (*DGAT1*), diacylglycerol acyltransferase 2 (*DGAT2*), and lipid phosphate phosphohydrolase 1 (*LPIN1*), which are downstream of *AGPAT2*. The expression levels of the *DGAT1* and *DGAT2* genes were significantly increased after the overexpression of lncRNA-46546 (Figure 8A, 8B). The expression level of the *LPIN1* gene did not significantly change in the overexpression group, but after the knockdown of lncRNA-46546, its expression level was significantly reduced (Figure 8C). These data demonstrated that lncRNA-46546 is involved in the lipid metabolism of ICP2 cells.

### lncRNA-46546 inhibits the proliferation of ICP2 cells

To further understand the function of lncRNA-46546, we used the CCK-8 assay to detect the proliferation of ICP2 cells at 24, 48, 72, and 96 h after the overexpression and knockdown of lncRNA-46546, and ddH_2_O served as a blank control. Each group included three replicates. The results showed that the proliferation of ICP2 cells was significantly inhibited after the transfection of pcDNA3.1(+)-46546 and decreased gradually beginning at 48 h (Figure 8D). After the knockdown of lncRNA-46546, the proliferation of ICP2 cells was not significantly affected but was lower than that in the blank control group (Figure 8E). In summary, these data indicated that lncRNA-46546 has an inhibitory effect on cell proliferation.

## DISCUSSION

In recent years, studies have shown that lncRNAs are widely distributed in animals, and lncRNAs present higher space-time and tissue specificity than coding genes and are less conserved among species [22]; these characteristics increase the difficulty of lncRNA research, but the use of RNA-seq facilitates lncRNA research. RNA-seq performed in humans has determined the molecular regulation mechanisms of fat accumulation between different groups of samples with different genetic backgrounds [23]. In this study, 96 and 42 differentially expressed lncRNAs were identified in the RCB vs CBB and RCL vs CBL comparisons, respectively. Interestingly, only 6 differentially expressed lncRNAs were shared between the two comparisons, possibly because lncRNAs show strong tissue specificity, consistent with the results of previous studies [24].

Numerous studies have shown that lncRNAs can regulate not only the expression of neighboring protein-coding genes through cis-acting mechanisms but also the expression of genes located on other chromosomes through trans-acting mechanisms [25]. In this study, 566 cis and 42 trans candidate target genes were predicted from 132 differentially expressed lncRNAs. Some of the candidate target genes were significantly enriched in GO terms associated with fat metabolism and have been previously investigated and reported; these genes present functions such as inhibiting the expression of the *NOTCH1* gene, which increases fatty acid oxidation in hepatocytes and reduces IMF deposition in the liver [26]. The activation of *NOTCH1* gene expression promotes the proliferation of preadipocytes [27]. The overexpression of *Id3* inhibits adiponectin and the differentiation of preadipocytes [28]. *ACSBG2* and *AGPAT2* have been reported to be involved in the lipid metabolism of chickens [29]. KEGG pathway analysis revealed the enrichment of *NOTCH1* in the NOTCH signaling pathway, *Id3* in the transforming growth factor β signaling pathway, *ACSBG2* in the *PPAR* signaling pathway, and *AGPAT2* in the glycerolipid metabolism, glycerophospholipid metabolism and metabolic pathways, and some of these pathways are considered classic lipid metabolism signaling pathways.

According to the above results, we selected the differen tially expressed lncRNA-46546 among the many differentially expressed lncRNAs for further functional research. The candidate target gene *AGPAT2* is a key rate-limiting enzyme in TG biosynthesis in adipocytes [30]. It belongs to the glycerol triphosphate pathway of de novo TG biosynthesis. In the TG synthesis pathway, glycerol-3-phosphate acyltransferase, mitochondrial (*GPAM*), *AGPAT2*, *LPINs* and other genes are involved in the enzymatic reactions of lysophospholipid acid (LPA), phospholipic acid (PA), and glycerol diester (DG) successively generated from glycerol triphosphate (G3P) [31]. These are important genes that regulate TG synthesis. The only enzyme that catalyzes the last step of TG synthesis is diacylglycerol acyltransferases (*DGATs*) [32]. A large number of studies show that congenital generalized lipodystrophy is caused by a lack of the *AGPAT2* gene, indicating that the *AGPAT2* gene is closely related to fat metabolism [33]. Studies have shown that *LPINs* and *DGATs* are also involved in fat metabolism and play important roles in the synthesis of TGs [34]. The results of this study showed that overexpression of lncRNA-46546 regulated and promoted the expression of downstream potential cis-target gene *AGPAT2*. The expression levels of *DGATS* and lipid phosphate phosphohydrolase (*LPINS*) were also correlated with *AGPAT2*. The expression of *AGPAT2* affected the generation of LPA and PA. Therefore, the expression levels of downstream *LPINS* and *DGATS* change correspondingly, promoting the transformation of LPA and PA to DG and TG. We speculate that the expression of the *AGPAT2* gene and its downstream genes in the pathway is also promoted by other factors (such as *HIF-1* and seipin proteins). *HIF-1* directly regulates the expression of the *AGPAT2* gene [35], and seipin and the *AGPAT2* gene can interact during early adipogenesis and potentiate the activity of adipogenic enzymes [36]. These factors need to be farther verified.

According to our results, lipid deposition and TG synthesis were promoted after the overexpression of lncRNA-46546 in ICP2 cells. The variation trend of intracellular and extracellular TG contents was consistent with the variation trend of the expression levels of *AGPAT2* and its downstream *DGAT* genes. Because the increased expression level of lncRNA-46546 affects the expression level of its target gene *AGPAT2*, affecting the generation of LPA. Finally, TG content in cells changed accordingly. In further studies, we found that after the overexpression of lncRNA-46546, the expression of *PPARγ*, *C/EBPα*, *FAS*, *SREBP1*, and *FABP4* were also significantly increased. After knockdown, the expression levels of *PPARγ* and *SREBP1* genes were significantly decreased. These results further confirm that lncRNA-46546 affects lipid metabolism and TG production. *PPARγ* and *C/EBPα* play important roles in early adipocyte differentiation [37]. *PPARγ* is a key regulator of adipogenesis, a necessary and sufficient condition for adipogenesis. So far, no factor has been found that can induce fat formation in the absence of *PPARγ* [38]. Recent studies have revealed that *PPARγ* and *C/EBPα* target genes are co-localized. *PPARγ - C*/*EBPα* positive feedback pathway enables pluripotent cells to differentiate into adipocytes [39,40]. Ramanathan et al [33] found that when the expression of the *AGPAT2* gene was knocked down, the expression of the PPARγ and C/EBPα genes was inhibited, and cell TG synthesis was also inhibited; Subauste et al [41] confirmed these results. Thus, an association clearly exists between the expression of the *AGPAT2*, *PPARγ*, and *C/EBPα* genes, but not with *FAS*, *SREBP1*, and *FABP4* genes. Our results also prove this finding. Finally, we detected the effect of lncRNA-46546 on the proliferation of ICP2 cells, and the results showed that overexpression of lncRNA-46546 inhibited cell proliferation; in contrast, its knockdown had no significant effect on cell proliferation, but the number of cells was lower than that in the blank control group. Knocking down the expression of the *AGPAT2* gene has been reported to lead to the accumulation of lysophosphatidic acid in cells, and excessive accumulation of lysophosphatidic acid can promote the proliferation of preadipocytes [42]. In this study, overexpression of lncRNA46546 led to up-regulation of *AGPAT2* expression, resulting in decreased accumulation of intracellular lysophosphatidic acid, thus inhibiting proliferation of preadipocytes.

In summary, our study screened out a highly differentially expressed lncRNA-46546 in chickens with two different genetic backgrounds. Functional verification at ICP2 cell level showed that LncRNA46546 promoted cell lipid deposition and TG synthesis by regulating its potential cis-target gene *GAPAT2*, and it can inhibit the proliferation of ICP2 cell. This study identified the molecular function of a lncRNA and provides good ideas for further exploring IMF deposition in early chicken embryo development to improve the meat quality of chickens and enhance molecular breeding.

## Figures and Tables

**Table 1:** Primers used for rapid amplification of cDNA ends polymerase chain reaction and vector construction

Primer name	Primer sequence (5′-3′)
lncRNA-46546-5′RACE-outer	GATTACGCCAAGCTTTGCCAGAAGAGGAGTGAGGTAGGAA
lncRNA-46546-5′RACE-inner	GATTACGCCAAGCTTAGATTACACGCACTCTCATTGGCTG
lncRNA-46546-3′RACE-outer	GATTACGCCAAGCTTGAGCAAACGAGGAAGTCTCGCTGCCTG
lncRNA-46546-3′RACE-inner	GATTACGCCAAGCTTCCGAAGAGAGAGGCATGTAGTGTGG
lncRNA-46546-5 (*KpnI*)	**CGGGGTACC**TCTATATAAGCGGAAGTCGGGAGGC
lncRNA-46546-3 (*XhoI*)	**CCGCTCGAG**GTATTGAAGAGAACTAGCTCAGACA

Underlined sequences represent the connection recognition sites, and sequences in bold represent the enzyme cutting sites.

RACE, rapid amplification of cDNA ends; PCR, polymerase chain reaction.

**Table 2:** siRNAs sequences used for RNA interference

siRNA name	Sequence (5′-3′)
siRNA-1	GCUGAGGACUUCCAUCUUATT
siRNA-2	GCGUUAUGAGUGUGAAAUATT
siRNA-3	GGGUAAGUCUGAAUCUCAATT
siRNA-NC	UUCUUCGAACGUGUCACGUTT

**Table 3:** Information on lncRNA-46545 and AGPAT2 expression from RNA-seq analysis

Gene ID	RCB FPKM	CBB FPKM	log_2_(fold change)	p-value	Q-value
*ENSGALT00000046546*	22.5375	13.7933	0.70835	0.016	0.039
*AGPAT2*	43.3841	32.7063	0.40759	0.00005	0.045

*AGPAT2*,1-acylglycerol-3-phosphate-O-acyltransferase 2; RCB, rose-crown chicken breast muscles; CBB, Cobb broiler chicken breast muscles; FPKM, fragments per kilobase million.

## SUPPLEMENTARY MATERIAL

Sequence data from this article have been deposited with the GenBank Data Libraries under Accession Nos. SRR12637775, SRR12637776, SRR12637777, SRR12637778, SRR12637779, SRR12637780, SRR12637781, SRR12637782, SRR12637783, SRR12637784, SRR12637785, SRR12637786. Novel lncRNA Sequence files obtained by sequencing have been uploaded to Sequence Read Archive Nos. SRR14253836.
