# Supplementary material for: Screening and functional validation of lipid metabolism-related lncRNA-46546 based on the transcriptome analysis of early embryonic muscle tissue in chicken
Source: Anim Biosci. 2022 Jan 21;36(2):175–90. doi: 10.5713/ab.21.0440 (PMC9834732; doi:10.5713/ab.21.0440)
Supplement: Supplementary file 1 [file ab-21-0440-suppl1.pdf]

Fig. S1. Introduction of Rose-crown chicken

Rosecrown chicken grows in Tianshan pastures, north of Xinjiang, China. Adult chicken has a bright, eye-catching and huge rose-shaped crown which is usually above 100 g. It is a rare and high-quality germ-plasm resource as the kind of broilers in China. There are four types based on color: black, white, red and the colour of a reed flower. Characteristics such as cold tolerance, adaptability, resistance is very excellent, and also it has great ornamental and simultaneously edible value due to its delicious meat. The weight of a rooster could be 2284±196 g at about 120 days, compared that the hen could be 1895±190 g. The age at first egg of hen is about 150 days and the egg production of one hen could be reached for 203~228 at the age of 66 weeks. The IMF (Intramuscular Fat) of adult rooster is 4.75±0.18% and 3.74±0.2% in leg and breast respectively, compared that the adult hen is 4.66±0.09% and 3.65±0.21%.

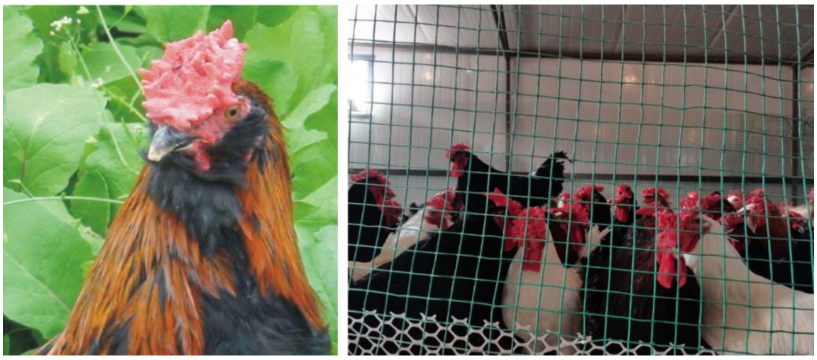

Fig.S1A. The cockscomb shape of Rose-crown chicken.

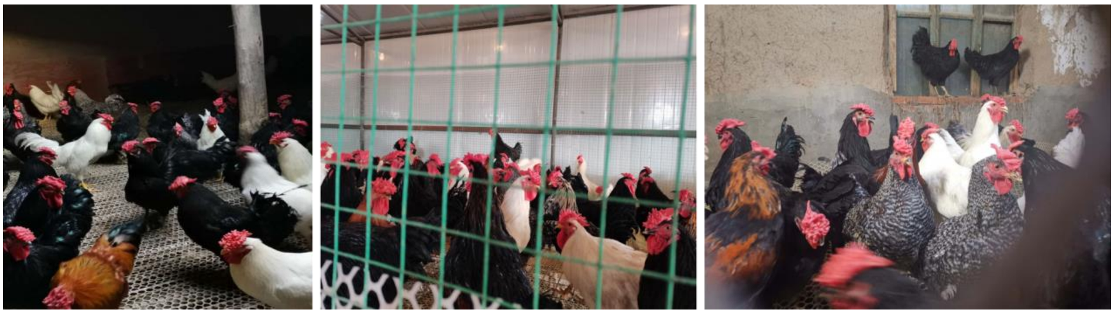

Fig.1B. The rooster group of Rose-crown chicken.

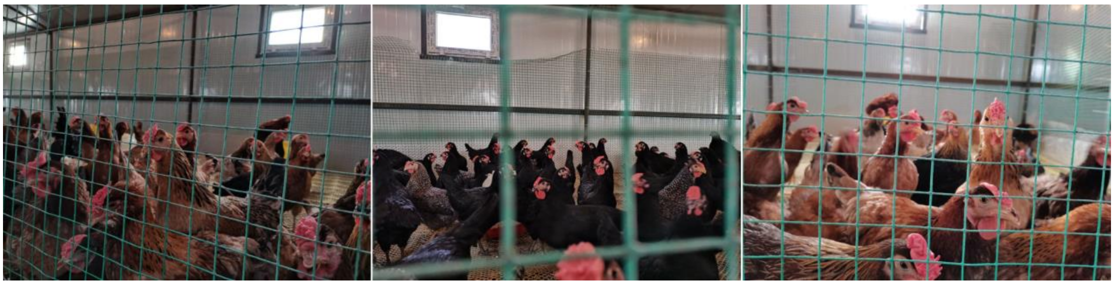

Fig.S1C. The hen group of Rose-crown chicken.

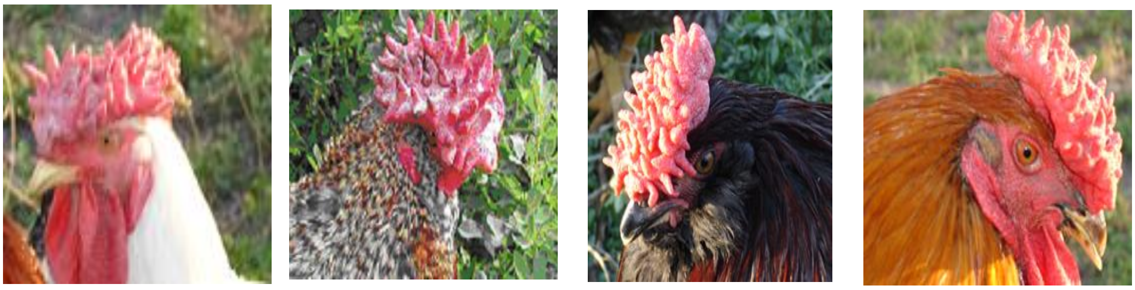

Fig.S1D. The feather colors of the Rose-crown chicken are white, reed, black and red from left to right.
