# Supplementary material for: Screening and functional validation of lipid metabolism-related lncRNA-46546 based on the transcriptome analysis of early embryonic muscle tissue in chicken
Source: Anim Biosci. 2022 Jan 21;36(2):175–90. doi: 10.5713/ab.21.0440 (PMC9834732; doi:10.5713/ab.21.0440)
Supplement: Supplementary file 2 [file ab-21-0440-suppl2.pdf]

Fig. S2. Validation of RNA-seq results by qRT-PCR.

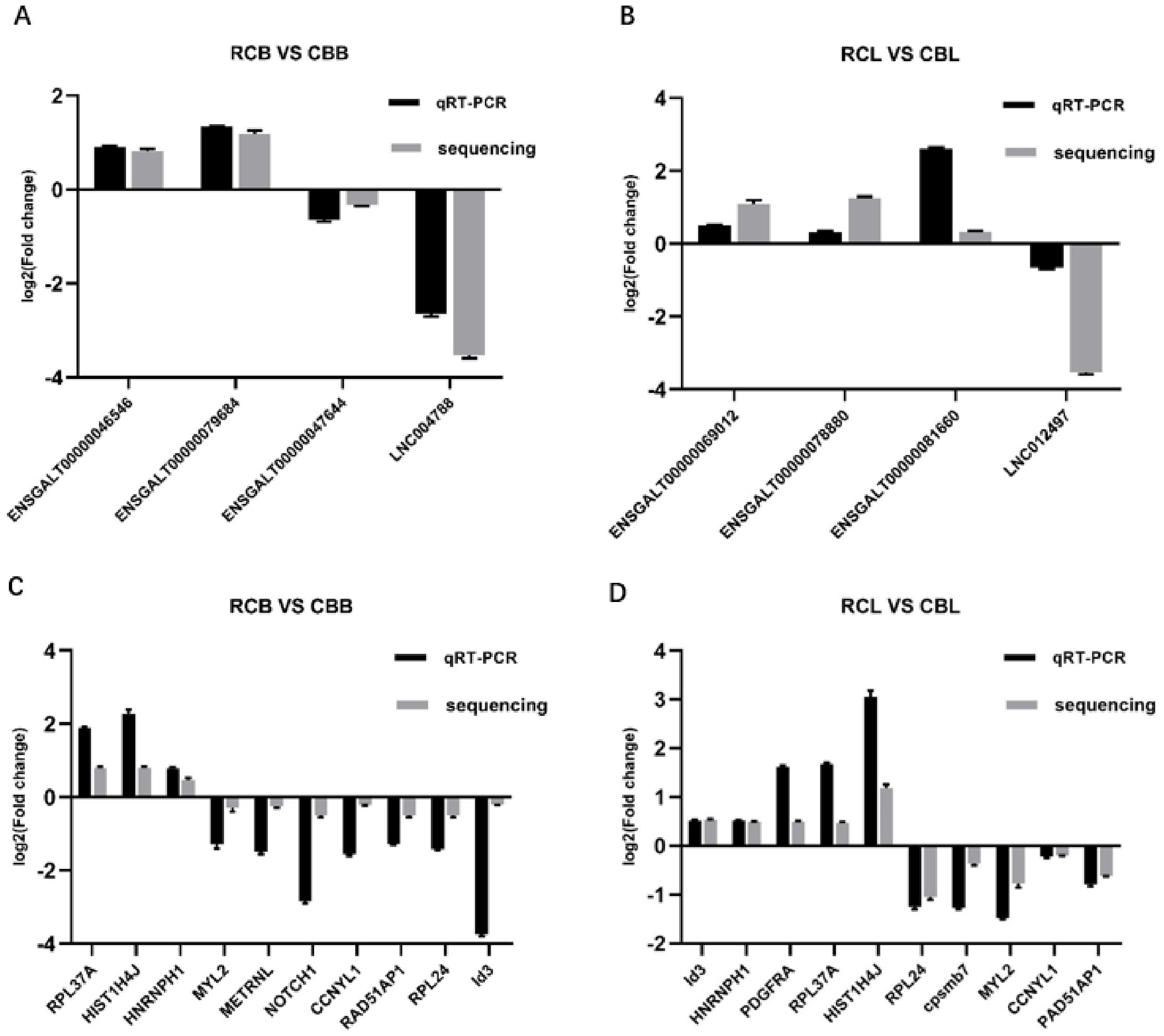

Fig. S2. Validation of RNA-seq results by qRT-PCR. (A) qRT-PCR validation of 4 lncRNAs between the RCB and CBB groups. (B) qRT-PCR validation of 4 lncRNAs between the RCL and CBL groups. (C) qRT-PCR validation of 10 mRNAs between the RCB and CBB groups. (D) qRT-PCR validation of 10 mRNAs between the RCL and CBL groups.
