# Supplementary material for: Screening and functional validation of lipid metabolism-related lncRNA-46546 based on the transcriptome analysis of early embryonic muscle tissue in chicken
Source: Anim Biosci. 2022 Jan 21;36(2):175–90. doi: 10.5713/ab.21.0440 (PMC9834732; doi:10.5713/ab.21.0440)
Supplement: Supplementary file 3 [file ab-21-0440-suppl3.pdf]

Fig. S3. Electrophoresis of RACE PCR products for lncRNA-ENSGALT000000 46546.

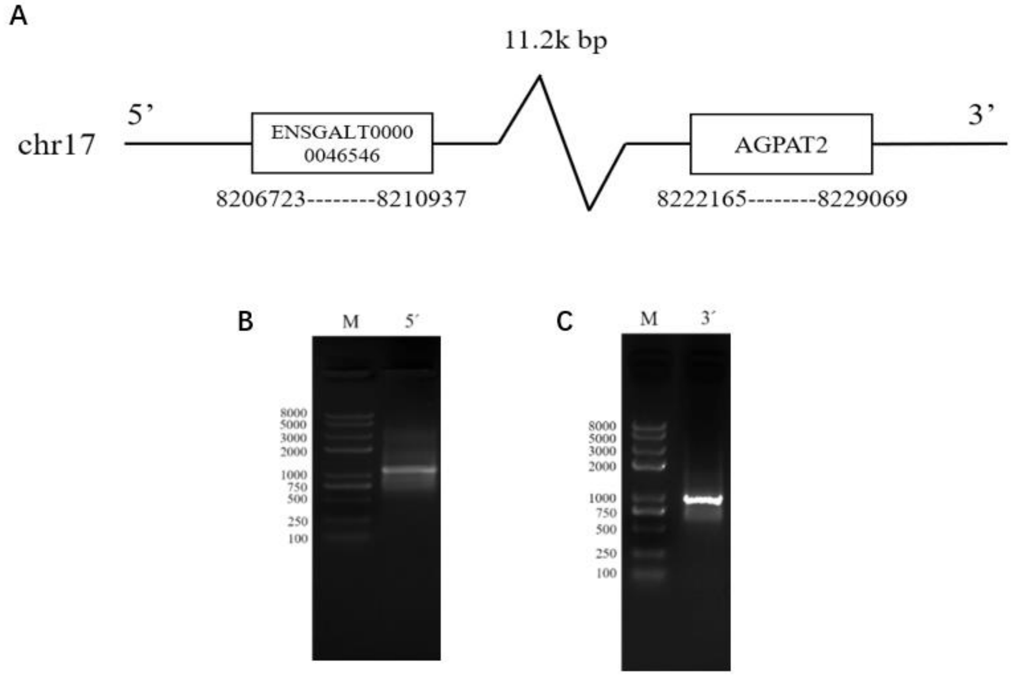

Fig. S3. Electrophoresis of RACE PCR products for lncRNA-ENSGALT00000046546. (**A**) lncRNA-46546 is located 11.2 k-bp upstream of the *AGPAT2* gene. **(B)** Electrophoresis of 5´ RACE PCR products to identify the 5´ portion of lncRNA-46546. **(C)** Electrophoresis of 3´ RACE PCR products to identify the 3´ portion of lncRNA-46546.
