# Supplementary material for: Screening and functional validation of lipid metabolism-related lncRNA-46546 based on the transcriptome analysis of early embryonic muscle tissue in chicken
Source: Anim Biosci. 2022 Jan 21;36(2):175–90. doi: 10.5713/ab.21.0440 (PMC9834732; doi:10.5713/ab.21.0440)
Supplement: Supplementary file 4 [file ab-21-0440-suppl4.pdf]

Fig. S4. sequence information of LncRNA-ENSGALT00000046546

```
ENSGALT00000046546.txt  ....................................................................................................     0
5'-RACE-PCR.txt         CTGTCCTTCTTCGCTATTACGCCCAGCCTGGCGAAAAGGAGGGATGTGCTGCAAGGCCGATTAAGTTGGGTAACGCCAGGGGTTTTCCCAGTCACGACGT   100
Consensus

ENSGALT00000046546.txt  .....................................TCTATA...TAAGCGGAAGTCGGGAGGCGGAGC......GGTCTTTCGGCGTGGCGCTCGGCG    54
5'-RACE-PCR.txt         TGTAAAACGACGGCCCAGTGAATTCCGAAAACGACTCACTATAGGGCAAGCAGTGGTATCAACGCAGAGTACATGGGGCTTTTCGGCGTGGCGCTCGGCG   200
Consensus                                                     ctata    aagc g  gt    a gc gag       gg  tttcggcgtggcgctcggcg

ENSGALT00000046546.txt  GGTGTTGGGTGTAGGCTGAGCTCGTTGTGAAGAGCAAACGAGGAAGTCTCGCTGCCTGCCCGCCCGCTCCTCTCCTGCCTTCAGGCTGAGAGGGCTCCCC   154
5'-RACE-PCR.txt         GGTGTTGGGTGTAGGCTGAGCTCGTTGTGAAGAGCAAACGAGGAAGTCTCGCTGCCTGCCCGCCCGCTCCTCTCCTGCCTTCAGGCTGAGAGGGCTCCCC   300
Consensus               ggtgttgggtgtaggctgagctcgttgtgaagagcaaacgaggaagtctcgctgcctgcccgcccgctcctctcctgccttcaggctgagagggctcccc

ENSGALT00000046546.txt  GCGGTGCCTTCCGGTGAAGCCGTGCCTTGCTGGCCGCTGTTCGCCGTCTCAGGCGCGGGCGCTATGCTGCAGCCGGGCCTCGGCTCTGGGATGGGCCGCG   254
5'-RACE-PCR.txt         GCGGTGCCTTCCGGTGAAGCCGTGCCTTGCTGGCCGCTGTTCGCCGTCTCAGGCGCGGGCGCTATGCTGCAGCCGGGCCTCGGCTCTGGGATGGGCCGCG   400
Consensus               gcggtgccttccggtgaagccgtgccttgctggccgctgttcgccgtctcaggcgcgggcgctatgctgcagccgggcctcggctctgggatgggccgcg

ENSGALT00000046546.txt  ACGGCGGCGGCCTCTGCCGTTTGTTGTCCGAGGCCGAGCGAGGGAAGGCGGGGTCGTGAGGGTACGGCCGCGGCCTGTAGGCTCGTATGGCGGAGGACGA   354
5'-RACE-PCR.txt         ACGGCGGCGGCCTCTGCCGTTTGTTGTCCGAGGCCGAGCGAGGGAAGGCGGGGTCGTGAGGGTACGGCCGCGGCCTGTAGGCTCGTATGGCGGAGGACGA   500
Consensus               acggcggcggcctctgccgtttgttgtccgaggccgagcgagggaaggcggggtcgtgagggtacggccgcggcctgtaggctcgtatggcggaggacga

ENSGALT00000046546.txt  GGAGGAGAAAAACAAGACAAAGGGTGGGATGAAAAAGATTTTCCATCTTGAAAAACCGAAGAGAGAGGCATGTAGTGTGGCAGACTCCAGAGGTCTCAAA   454
5'-RACE-PCR.txt         GGAGGAGAAAAACAAGACAAAGGGTGGGATGAAAAAGATTTTCCATCTTGAAAAACCGAAGAGAGAGGCATGTAGTGTGGCAGACTCCAGAGGTCTCAAA   600
Consensus               ggaggagaaaaacaagacaaagggtgggatgaaaaagattttccatcttgaaaaaccgaagagagaggcatgtagtgtggcagactccagaggtctcaaa

ENSGALT00000046546.txt  AGCCCGAGGAGGAAAAATTGAAGATACTTTTTCTTCATGCAGTTACACGTGAGGACAAGTCTGAGTTGTTGTGCAGTTCCCTTCAGATGTTGAAGATCAT   554
5'-RACE-PCR.txt         AGCCCGAGGAGGAAAAATTGAAGATACTTTTTCTTCATGCAGTTACACGTGAGGACAAGTCTGAGTTGTTGTGCAGTTCCCTTCAGATGTTGAAGATCAT   700
Consensus               agcccgaggaggaaaaattgaagatactttttcttcatgcagttacacgtgaggacaagtctgagttgttgtgcagttcccttcagatgttgaagatcat

ENSGALT00000046546.txt  GCTTGAATTCTAGAATTTCTAGCAAGCCTGGCTACAGGAAAAGAAATGGTTTAGCATTTGTGCTTCAAGATGCTGAGGACTTCCATCTTATTCACACATG   654
5'-RACE-PCR.txt         GCTTGAATTCTAGAATTTCTAGCAAGCCTGGCTACAGGAAAAGAAATGGTTTAGCATTTGTGCTTCAAGATGCTGAGGACTTCCATCTTATTCACACATG   800
Consensus               gcttgaattctagaatttctagcaagcctggctacaggaaaagaaatggtttagcatttgtgcttcaagatgctgaggacttccatcttattcacacatg

ENSGALT00000046546.txt  GATTCAAGCAAGCTGCAGCCAATGAGAGTGCGTGTAATCTTTCCTTCCTACAAGTAATCTCAGCTTGTGCTGTTTTCACACCCATGTTCTGAAGCACTTG   754
5'-RACE-PCR.txt         GATTCAAGCAAGCTGCAGCCAATGAGAGTGCGTGTAATCTTTCCTTCCTACAAGTAATCTCAGCTTGTGCTGTTTTCACACCCATGTTCTGAAGCACTTG   900
Consensus               gattcaagcaagctgcagccaatgagagtgcgtgtaatctttccttcctacaagtaatctcagcttgtgctgttttcacacccatgttctgaagcacttg

ENSGALT00000046546.txt  TTTTGACCACGTTTACCACGTGGCTAAGAAGGTTTAAAAAGAAGAACACTAAGTATGCTATCTGCAAGTCTGAAATAATGCTTTTTAAACATCCTAGGAT   854
5'-RACE-PCR.txt         TTTTGACCACGTTTACCACGTGGCTAAGAAGGTTTAAAAAGAAGAACACTAAGTATGCTATCTGCAAGTCTGAAATAATGCTTTTTAAACATCCTAGGAT  1000
Consensus               ttttgaccacgtttaccacgtggctaagaaggtttaaaaagaagaacactaagtatgctatctgcaagtctgaaataatgctttttaaacatcctaggat

ENSGALT00000046546.txt  CTAGGATGTTGTTGTCTGTGGATATGAGAATTGTGGGGTTTTCTTAACCTTAAACAATGAAATGTGGAAGCTTGCTTCCTACCTCACTCCTCTTCTGGCA   954
5'-RACE-PCR.txt         CTAGGATGTTGTTGTCTGTGGATATGAGAATTGTGGGGTTTTCTTAACCTTAAACAATGAAATGTGGAAGCTTGCTTCCTACCTCACTCCTCTTCTGGCA  1100
Consensus               ctaggatgttgttgtctgtggatatgagaattgtggggttttcttaaccttaaacaatgaaatgtggaagcttgcttcctacctcactcctcttctggca

ENSGALT00000046546.txt  CTTAAATCTTTTACACTGCTAAAGCAGCAGCGTCTGTTAGTGTTTGGTTGTCAGTCTTTCAGTGTTCTGGCTCTTGCTCCTTTTCCAAC...TAATGTGG  1051
5'-RACE-PCR.txt         .....AAGCCAAGCGCAATCATGGTCATAGCTGTTTCCTGTGTGAAATTGTTATCCGCTCACAATTTCCACACAACATACGAGCCGGAAGCATAAAGTGT  1195
Consensus                    a       c c    a  g    agc   t    gtgt    ttgt a  c  tca   tt    c c    t c    c  a    taa gtg

ENSGALT00000046546.txt  AAAG.TTGGGAGGAGGCGTTATGAGTGTGAAATAGCTTTGCCTTGAGTCATTTCTCTTCCATATTGTACTGTAGCTCGTAAACCATAATCACTTGTTTGA  1150
5'-RACE-PCR.txt         AAAGGCCTGGGGGTTGCCTAATGAGTG.........................................................................  1222
Consensus               aaag    gg gg  gc t atgagtg

ENSGALT00000046546.txt  AAACACTTGTCCATTGTATTAGTGACGAATGAAACTTGGTGCTCTTGGGATGTGTGAGTCATGGGTAAGTCTGAATCTCAAAGTGTCTGTCCAGTGCTGT  1250
5'-RACE-PCR.txt         ....................................................................................................  1222
Consensus

ENSGALT00000046546.txt  TAATGGGGTGTGCAGAGCAGTAACATGGTAACATCGTGGGTTTTGTCAAAAGTCTTTATTTGAATAAAGTGTCTGAGCTAGTTCTC                1336
5'-RACE-PCR.txt         ......................................................................................                1222
Consensus
```

Fig.S4A. LncRNA-ENSGALT00000046546-5 'RACE PCR sequence alignment results

```
ENSGALT00000046546.txt   TCTATATAAGCGGAAGTCGGGAGGCGGAGCGGTCTTTCGGCGTGGCGCTCGGCGGGTGTTGGGTGTAGGCTGAGCTCGTTGTGAAGAGCAAACGAGGAAG    100
3'-RACE-PCR.txtRC        ....................................................................................................      0
Consensus

ENSGALT00000046546.txt   TCTCGCTGCCTGCCCGCCCGCTCCTCTCCTGCCTTCAGGCTGAGAGGGCTCCCCGCGGTGCCTTCCGGTGAAGCCGTGCCTTGCTGGCCGCTGTTCGCCG    200
3'-RACE-PCR.txtRC        ....................................................................................................      0
Consensus

ENSGALT00000046546.txt   TCTCAGGCGCGGGCGCTATGCTGCAGCCGGGCCTCGGCTCTGGGATGGGCCGCGACGGCGGCGGCCTCTGCCGTTTGTTGTCCGAGGCCGAGCGAGGGAA    300
3'-RACE-PCR.txtRC        ....................................................................................................      0
Consensus

ENSGALT00000046546.txt   GGCGGGGTCGTGAGGGTACGGCCGCGGCCTGTAGGCTCGTATGGCGGAGGACGAGGAGGAGAAAAACAAGACAAAGGGTGGG.ATGAAAAAGATTTTCCA    399
3'-RACE-PCR.txtRC        ............ACACCTTTTATGCTTCCGGCTCGTATGTTGTGTGGAATTGTGAGCGGATAACAATTTCACCCAGAACAGCTATGACCATGATACGCTG     88
Consensus                            a          gc  cc g   g   gt   g gga       g gga aa aa    ac  ag    g  atga  a gat   c

ENSGALT00000046546.txt   TCTTGAAAAACCGAAGAGAGAGGCATGTAGTG.TGGCAGACTCC.AGAGGTCTCAAAAGCCCGAGGAGGAAAAATTGAAGATACTTTTTCTTCATGCAGT    497
3'-RACE-PCR.txtRC        GCTTG......TGGAGAGAGAGGCATGTAGTGGTGGCAGACTCCCAGAGGTCTCAAAAGCCCGAGGAGGAAAAATTGAAGATACTTTTTCTTCATGCAGT    182
Consensus                 cttg       g agagagaggcatgtagtg tggcagactcc agaggtctcaaaagcccgaggaggaaaaattgaagatactttttcttcatgcagt

ENSGALT00000046546.txt   TACACGTGAGGACAAGTCTGAGTTGTTGTGCAGTTCCCTTCAGATGTTGAAGATCATGCTTGAATTCTAGAATTTCTAGCAAGCCTGGCTACAGGAAAAG    597
3'-RACE-PCR.txtRC        TACACGTGAGGACAAGTCTGAGTTGTTGTGCAGTTCCCTTCAGATGTTGAAGATCATGCTTGAATTCTAGAATTTCTAGCAAGCCTGGCTACAGGAAAAG    282
Consensus                tacacgtgaggacaagtctgagttgttgtgcagttcccttcagatgttgaagatcatgcttgaattctagaatttctagcaagcctggctacaggaaaag

ENSGALT00000046546.txt   AAATGGTTTAGCATTTGTGCTTCAAGATGCTGAGGACTTCCATCTTATTCACACATGGATTCAAGCAAGCTGCAGCCAATGAGAGTGCGTGTAATCTTTC    697
3'-RACE-PCR.txtRC        AAATGGTTTAGCATTTGTGCTTCAAGATGCTGAGGACTTCCATCTTATTCACACATGGATTCAAGCAAGCTGCAGCCAATGAGAGTGCGTGTAATCTTTC    382
Consensus                aaatggtttagcatttgtgcttcaagatgctgaggacttccatcttattcacacatggattcaagcaagctgcagccaatgagagtgcgtgtaatctttc

ENSGALT00000046546.txt   CTTCCTACAAGTAATCTCAGCTTGTGCTGTTTTCACACCCATGTTCTGAAGCACTTGTTTTGACCACGTTTACCACGTGGCTAAGAAGGTTTAAAAAGAA    797
3'-RACE-PCR.txtRC        CTTCCTACAAGTAATCTCAGCTTGTGCTGTTTTCACACCCATGTTCTGAAGCACTTGTTTTGACCACGTTTACCACGTGGCTAA.AAGGTTTAAAAAGAA    481
Consensus                cttcctacaagtaatctcagcttgtgctgttttcacacccatgttctgaagcacttgttttgaccacgtttaccacgtggctaa aaggtttaaaaagaa

ENSGALT00000046546.txt   GAACACTAAGTATGCTATCTGCAAGTCTGAAATAATGCTTTTTAAACATCCTAGGATCTAGGATGTTGTTGTCTGTGGATATGAGAATTGTGGGGTTTTC    897
3'-RACE-PCR.txtRC        GAACACTAAGTATGCTATCTGCAAGTCTGAAATAATGCTTTTTAAACATCCTAGGATTTAGGATGTTGTTGTCTGTGGATATGAGAATTGTGGGGTTTTC    581
Consensus                gaacactaagtatgctatctgcaagtctgaaataatgctttttaaacatcctaggat taggatgttgttgtctgtggatatgagaattgtggggttttc

ENSGALT00000046546.txt   TTAACCTTAAACAATGAAATGTGGAAGCTTGCTTCCTACCTCACTCCTCTTCTGGCACTTAAATCTTTTACACTGCTAAAGCAGCAGCGTCTGTTAGTGT    997
3'-RACE-PCR.txtRC        TTAACCTTAAACAATGAAATGTGGAAGCTTGCTTCCTACCCCACTCCTCTTCTGGCACTTAAATCTTTTACACTGCTAAAGCAGCAGCGTCTGTTCGTGT    681
Consensus                ttaaccttaaacaatgaaatgtggaagcttgcttcctacc cactcctcttctggcacttaaatcttttacactgctaaagcagcagcgtctgtt gtgt

ENSGALT00000046546.txt   TTGGTTGTCAGTCTTTCAGTGTTCTGGCTCTTGCTCCTTTTCCAACTAATGTGGAAAGTTGGGAGGAGGCGTTATGAGTGTGAAATAGCTTTGCCTTGAG   1097
3'-RACE-PCR.txtRC        TTGGTTGTCAGTCTTTCAGTGTTCTGGCTCTTGCTCCTTTTCCAACTAATGTGGAAAGTTGGGAGGAGGCGTTATGAGTGTGAAATAGCTTTGCCTTGAG    781
Consensus                ttggttgtcagtctttcagtgttctggctcttgctccttttccaactaatgtggaaagttgggaggaggcgttatgagtgtgaaatagctttgccttgag

ENSGALT00000046546.txt   TCATTTCTCTTCCATATTGTACTGTAGCTCGTAAACCATAATCACTTGTTTGAAAACACTTGTCCATTGTATTAGTGACGAATGAAACTTGGTGCTCTTG   1197
3'-RACE-PCR.txtRC        TCATTTTTCTTCCATATTGTACTGTAGCTCGTAAACCATAATCACTTGTTTGAAAACACTTGTCCATTGTATTAGTGACGAATGACACTTGGTGCTCTTG    881
Consensus                tcattt tcttccatattgtactgtagctcgtaaaccataatcacttgtttgaaaacacttgtccattgtattagtgacgaatga acttggtgctcttg

ENSGALT00000046546.txt   GGATGTGTGAGTCATGGGTAAGTCTGAATCTCAAAGTGTCTGTCCAGTGCTGTTAATGGGGTGTGCAGAGCAGTAACATGGTAACATCGTGGGTTTTGTC   1297
3'-RACE-PCR.txtRC        GGATGTGTGAGTCATGGGTAAGTCTGAATCTCAGAGTGTCTGTCCAGTGCTGTTAATGGGGTGTGCAGAGCAGTAACATGGTAACATCGTGGGTTTTGTC    981
Consensus                ggatgtgtgagtcatgggtaagtctgaatctca agtgtctgtccagtgctgttaatggggtgtgcagagcagtaacatggtaacatcgtgggttttgtc

ENSGALT00000046546.txt   AAAAGTCTTTATTTGAATAAAGTGTCTGAGCTAGTTCTCT............................................................   1337
3'-RACE-PCR.txtRC        AAAAGTCTTTATTTGAATAAAGTGTCTGAGCTAGTTCTCTTCAATACAAAAAAAAAAAAAAAATAGAAAAAAAAAGTACTCTGCGTTGATACCACTGCTTG   1081
Consensus                aaaagtctttatttgaataaagtgtctgagctagttctct

ENSGALT00000046546.txt   ..................                                                                                     1337
3'-RACE-PCR.txtRC        CCCTATAGGAGTCGTGCC                                                                                     1099
Consensus
```

Fig.S4B. LncRNA-ENSGALT00000046546-3 'RACE PCR sequence alignment results

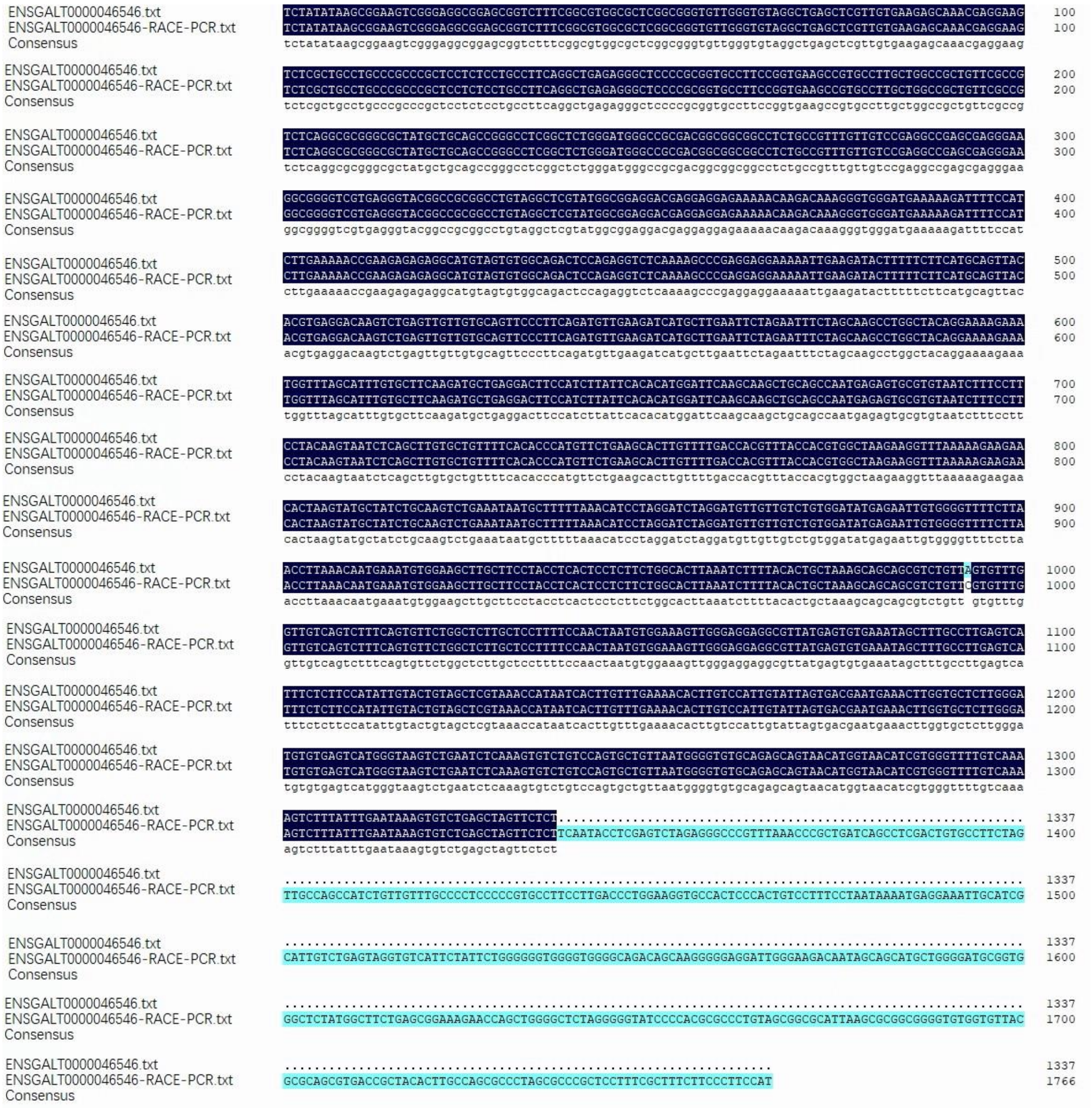

Fig.S4C. LncRNA-ENSGALT00000046546 full-length amplification sequence alignment results
