# Supplementary material for: Screening and functional validation of lipid metabolism-related lncRNA-46546 based on the transcriptome analysis of early embryonic muscle tissue in chicken
Source: Anim Biosci. 2022 Jan 21;36(2):175–90. doi: 10.5713/ab.21.0440 (PMC9834732; doi:10.5713/ab.21.0440)
Supplement: Supplementary file 5 [file ab-21-0440-suppl5.pdf]

File S1 Primer information for qRT-PCR

gene	Primer sequence (5´-3´)	Product length (bp)
FAS	F: CATCACCGTCTATCGGAACC<br>R: TCGTAGGCTCCTCCCATCC	125
LPL	F: GTCTGCTACCTGGTTCCGG<br>R: ATTCCTGTCACCGTCCACCC	104
SREBP1	F: CACCGAGGCTAAGCTCAACAA<br>R: CTTGAGGGACTTGCTCTTCTGC	139
FABP4	F: CAAGCCTAATTTAACTATCAGCATC<br>R: AGGTTCCCATCCACCACTTT	231
C/EBPα	F: ACGAGCACTCCATCGACATCAGC<br>R: GGGTCGAGCTTGCCGTCCAT	244
PPARγ	F: CCAACTCACTTATGGCTAT<br>R: CTGCTTTTCTTATGGATGC	169
AGPAT2	F: CAAAACCGTGGTCAAGTCC<br>R: CCCCGAGGTATGTGATGAG	221
GAPDH	F: TGGCATCCAAGGAGTGAGC<br>R: GGGAGACAGAAGGGAACAGAA	140
ENSGALT00000046546	F: AACAAGACAAAGGGTGGGA<br>R: TCAACATCTGAAGGGAACTG	183
ENSGALT00000079684	F: CTGCCGCTACGCTGAAGA<br>R: TTGCCACCACCTCCACTCG	103
ENSGALT00000047644	F: TGACCAGGTGTCGTAGCC<br>R: GCACGCACATAGGGACTG	159
ENSGALT00000069012	F: GTGGCGGCTTCAAGTCAA<br>R: GGCAGTCTCCTGTTGCGTAT	125
ENSGALT00000078880	F: CGAGAACAGGGGGAATGACC<br>R: GCTGAGCGTGTTTCGGTGC	171
ENSGALT00000081660	F: CGGCTTCCTCGTGTCCTTTG<br>R: CCGACTCCGATGGTTTCCTCT	161
LNC004788	F: GCAGAAACCAGAGCAGGACAC<br>R: CCGCCAAGTACCGTTAAGACG	142
LNC012497	F: TCACCCATTGGACTCAGCAC<br>R: TGAAGGGACAGCCCAGGAAC	388
	F: AATCAGCCAACACGCCAAGT	

RPL37A	R: CCAGGCACCACCAGCAACT	118
HIST1H4J	F: GCGACAACATCCAGGGCATCA<br>R: AACCGCCGAAGCCGTAGAGG	240
HNRNPH1	F: TCTGACCTCCAATGTGATGC<br>R: TGCCCTCCCTCGTGTAGAT	166
MYL2	F: GGCAAAGGGCTGAAATCTG<br>R: GGCTGTTATCCCGTTGAAGA	276
METRNL	F: GGTCTACCTCCGCTGTTCC<br>R: TGTGATCTTCCTGCTAATGTCC	292
NOTCH1	F: GACAGCATCGCCGCCTTCAC<br>R: CGTCCAGGTTGATCTCGCAGTTG	187
CCNYL1	F: TTCCCTGGTTTGGCTACTGA<br>R: GGATTCCTTCCTGCTGTGC	79
RAD51AP1	F: TGTGAAAGGGATTTGAACG<br>R: CCATTGCCTACAGCCATTC	135
RPL24	F: TCAGAGGGCTATTACTGGTGC<br>R: TGCTTAGGTGCTGCCTTTGT	186
Id3	F: CGCAGCAGCAACAACAAGAG<br>R: CTGATGGAGGAGGCGTTAGTGA	309
PDGFRA	F: AGATGACGGTTCGGAGGGT<br>R: CTAGCCAACCCAAAGTCGCA	174
cpsmb7	F: GAGGAAGAAGAAGCCAAACA<br>R: CATCATACGGACGGATAAAA	133
U6	F: CAAATTGGCTAAGCGGGCCT<br>R: CTAACAGCGTCGAGACTGCG	139
